# Preference for Safe Over Risky Options in Binge Eating

**DOI:** 10.3389/fnbeh.2016.00065

**Published:** 2016-03-31

**Authors:** Rémi Neveu, Elsa Fouragnan, Franck Barsumian, Edouard Carrier, Massimo Lai, Alain Nicolas, Dorine Neveu, Giorgio Coricelli

**Affiliations:** ^1^Neuroscience Research Center, CNRS, Université de LyonLyon, France; ^2^Praxis, Ville-la-GrandFrance; ^3^Institute of Psychology and Neurosciences, University of GlasgowGlasgow, Scotland, UK; ^4^Clinique Saint Vincent de PaulLyon, France; ^5^Clinique Lyon-Lumière, MeyzieuFrance; ^6^Hôpital du Vinatier, BronFrance; ^7^Université Montpellier 1, INSERM U 1058Montpellier, France; ^8^Department of Economics, University of Southern CaliforniaLos Angelès, CA, USA

**Keywords:** decision-making, risk, uncertainty, binge eating, bulimia nervosa, anorexia nervosa, cognitive control, loss aversion

## Abstract

Binge eating has been usually viewed as a loss of control and an impulsive behavior. But, little is known about the actual behavior of binging patients (prevalently women) in terms of basic decision-making under risk or under uncertainty. In healthy women, stressful cues bias behavior for safer options, raising the question of whether food cues that are perceived as threatening by binging patients may modulate patients’ behaviors towards safer options. A cross-sectional study was conducted with binging patients (20 bulimia nervosa (BN) and 23 anorexia nervosa binging (ANB) patients) and two control groups (22 non-binging restrictive (ANR) anorexia nervosa patients and 20 healthy participants), without any concomitant impulsive disorder. We assessed decisions under risk with a gambling task with known probabilities and decisions under uncertainty with the balloon analog risk taking task (BART) with unknown probabilities of winning, in three cued-conditions including neutral, binge food and stressful cues. In the gambling task, binging and ANR patients adopted similar safer attitudes and coherently elicited a higher aversion to losses when primed by food as compared to neutral cues. This held true for BN and ANR patients in the BART. After controlling for anxiety level, these safer attitudes in the food condition were similar to the ones under stress. In the BART, ANB patients exhibited a higher variability in their choices in the food compared to neutral condition. This higher variability was associated with higher difficulties to discard irrelevant information. All these results suggest that decision-making under risk and under uncertainty is not fundamentally altered in all these patients.

Binge eating episodes are periods of large food intake within short time. They are commonly viewed as a failure of the strict self-control over food/caloric intake exerted by patients with bulimia nervosa (BN), anorexia nervosa binging subtype (ANB) and eating disorders not otherwise specified (EDNOS; Fairburn and Harrison, 2003). Binge eating is associated with serious negative consequences, such as drop in self-esteem, strengthening of the strict control over food intake and negative physiological consequences of purging behaviors. At the time of the binge, these long-term negative consequences are neglected by patients who focus exclusively on the immediate appetitive outcome (i.e., binge foods; Boeka and Lokken, 2006). Therefore, binging patients would select more often more appetitive but probabilistic rather than safe but less appetitive options (Cavedini et al., 2004; Boeka and Lokken, 2006; Brand et al., 2007; Tchanturia et al., 2007; Brogan et al., 2010; Van den Eynde et al., 2012; Wu et al., 2013). However, this putative behavior is questionable. First, a lack in integrating long-term consequences in decisions is not specific of the binge. The reduced sensitivity to long-term negative consequences occurs also during the strict self-control over food intake as this latter involves banning appetitive foods which in turns facilitates binge occurrence (Fairburn and Harrison, 2003). Second, by violating their strict self-control over food intake, patients take the risk to engage in a binge that contradicts the large effort made to restrict foods that are usually eaten during binges (Fairburn and Harrison, 2003; Neveu et al., 2014). Third, binging patients recruit planning skills during binges to serve binge food restriction (Neveu et al., 2014). Fourth, a high level of anxiety, as elicited by binge foods in eating disorder patients (Neveu et al., 2014), leads to more conservative choices under risk and under uncertainty in women (Lighthall et al., 2009; Porcelli and Delgado, 2009; van den Bos et al., 2009; Starcke and Brand, 2012), who constitute 90% of eating disorder patients (Fairburn and Harrison, 2003). Thus, binging women should prefer, rather than refuse, choices for safer options in a binge context.

To date, studies investigating risky choices in binging patients have led to inconsistent results: some claim that binging patients choose more often risky options with small outcome expectancies (Brand et al., 2007; Tchanturia et al., 2007; Brogan et al., 2010) while other studies with larger sample sizes have not found any differences between binging patients and healthy controls (Cavedini et al., 2004; Van den Eynde et al., 2012; Wu et al., 2013). These discrepancies might be due to different levels of attention (Hare et al., 2011; Lim et al., 2011) and cognitive interferences such as preoccupations toward food and weight (Fairburn and Harrison, 2003; Starcke et al., 2011; Aïte et al., 2013) at assessment. These studies also did not investigate reaction time (Cavedini et al., 2004; Brand et al., 2007; Tchanturia et al., 2007; Brogan et al., 2010; Van den Eynde et al., 2012; Wu et al., 2013) which provides compelling information about the decision process (Milosavljevic et al., 2010). Moreover, participants were assessed without being primed and aroused by food, limiting conclusions about the decision-making processes involved in binges (Cavedini et al., 2004; Brand et al., 2007; Tchanturia et al., 2007; Brogan et al., 2010; Van den Eynde et al., 2012; Wu et al., 2013). A meta-analysis, performed on studies that did not involve any food condition, showed that the preference for risky options is stronger in patients with restrictive anorexia nervosa (ANR), who do not binge, than in binging patients, suggesting that such a preference is not specific to binge eating (Guillaume et al., 2015).

We investigated the decision-making process involved in risky situations in binging (BN and ANB) patients when primed by neutral, food and stressful cues and compared their performances to those of non-binging (ANR) patients and healthy participants. Situations of risk were characterized by the amount of information that was available to the decision maker with respect to the probability of an outcome to occur. Thus, decisions varied from situations where outcome probabilities were fully known (decision under risk) or unknown (decision under uncertainty). We specifically hypothesized that binging patients (BN and ANB) would make more conservative choices when primed by food cues as compared to neutral cues. Because foods elicit anxiety also in non-binging ANR patients (Neveu et al., 2014) and anxiety is associated with more conservative behaviors (Lighthall et al., 2009; Porcelli and Delgado, 2009; van den Bos et al., 2009; Starcke and Brand, 2012), ANR patients are likely to make safer choices in food than in neutral conditions as binging patients would do. However, this would not be the case for healthy participants. We also hypothesized that this safer behavior would be similar to the one observed in the stressful condition in these patients. Finally, we hypothesized that the differential behavior between food and neutral conditions in patients would be similar to the one of healthy controls in stressful compared to neutral conditions.

## Materials and Methods

### Study Design and Population

Women, aged 18–35 years, with a body mass index (BMI) < 25 kg/m^2^, were enrolled in a cross-sectional study between November 2010 and February 2011. Four groups were constituted: women free of any eating disorder, or with a current diagnosis of BN with or without purging behaviors, restrictive (ANR) or binging (ANB) anorexia nervosa (DSM-IV R criteria). Patients’ psychotropic medication was stabilized for more than 1 week in order to avoid interaction with neuropsychological performances (van Laar et al., 2002; Drueke et al., 2009). Exclusion criteria were: any addiction, histrionic personality disorder, psychotic disorder, dementia or mental retardation and the following impulsive disorders; antisocial personality disorder, attention deficit and hyperactivity disorder, borderline personality disorder, intermittent explosive disorder (DSM-IV R criteria). Controls were recruited through e-mail advertisements and patients in inpatient units specialized in the treatment of eating disorders (Lyon, Meyzieu, Ville-la-Grand, Vérargues; France). Participants were assessed using the structured clinical interview for DSM-IV R diagnoses. All participants provided a written informed consent before inclusion. The study was approved by the independent ethical committee, Comité de Protection des Personnes Sud Est IV.

### Assessment

To account for diurnal variations of binge occurrence across the day, participants were randomly allocated to morning or afternoon sessions (Smyth et al., 2009) with stratification by site and diagnosis.

#### Tasks

**Figure 1:**
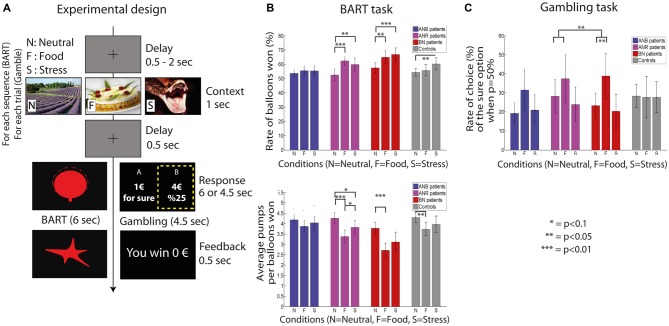
**Trial design in the balloon analog risk taking (BART) and gambling tasks (A), rate of balloons saved (in %) in BART and average number of pumps per balloon saved in BART (B), rate of choices of the gamble when the probability to win the gamble is 50% in the gambling task (C).** Anorexia Nervosa Restrictive (ANR) are ANR subtype patients, anorexia nervosa binging (ANB) are ANB subtype patients, bulimia nervosa (BN) are BN patients and controls are healthy participants. Mean and standard errors of the mean are reported. Note: In the BART, participants have to choose between inflating one more time the balloon or saving it. The response event is repeated until balloon explosion or participant’s choice to save the balloon. A sequence corresponds to all participants choices from the first display of a balloon until its saving or its explosion. In the gambling task, participants have to choose between one of the two options.

All participants performed a monetary gambling task and the balloon analog risk taking (BART) task (Lejuez et al., 2002; Figure 1A) in computerized protocols using Presentation (NeuroBehavioral Systems, release 14.2, Albany, CA, USA). The gambling task captures decision-making under risk whereas the BART captures decision making under uncertainty (Brand et al., 2006). These two tasks complemented the assessment of other cognitive control abilities for which results are reported elsewhere (Neveu et al., 2014). At the beginning of each of the two tasks, participants were trained over five trials at the gambling task and two balloons sequences at the BART to get used to the response buttons.

The gambling task assessed the ability to choose between a sure payoff and a gamble with explicit probabilities of winning. The gamble had two outcomes: a positive payoff when winning and 0€ otherwise. At each trial, the expected value of the gamble (i.e., probability of winning multiplied by winning payoff) was equal to the payoff of the safe choice which ranged from 1€ to 10€. The probability to win the gamble was set to 0.1, 0.25, 0.5, 0.75 or 0.9 and randomly assigned to each gamble in a way that the expected value could not be higher than 10€. In total, participants played 75 gambles. A feedback indicating the participant’s reward was displayed after each choice.

In the BART, participants had to choose on each trial between saving the ongoing balloon or pumping on to inflate the balloon by a fixed volume. The size of the balloon represents payoff when it is saved. However, the bigger the balloon is, the higher is the risk of exploding it with an additional inflate. Note that there might be several pumps, and so several trials, performed for the same balloon before saving it or making it explode. When the ongoing balloon is saved or has exploded, a new one is presented. The initial size of the balloon was the same for all balloons. Probability for the balloon to explode increased linearly by 9% at each inflate reaching 100% chances to explode at the 11th inflation. This probability was not provided to participants. For this task, a sequence represents the number of times participants made a decision until the balloon explodes or was saved. Payoff feedback was given only at the end of each sequence. Participants performed each balloon sequence 90 times.

Participants were told that one random trial would be selected at the end of the experiment for each task and the corresponding payoff would be added to a fixed 60€ received for their participation. In total, participants could improve their earning by 10€. This allowed maintaining the participant’s attention throughout the task.

In addition to these two tasks, participants performed the Simon task (Craft and Simon, 1970; i.e., the non-verbal Stroop task) which assesses the resistance to interference from outside stimuli, and the Go/No-go task (Newman et al., 1985), which assesses the ability to repeat (go trials) and inhibit (no-go trials) an automated behavior.

#### Preconditioning

Each task was assessed in three preconditioning situations: food, stressful or neutral conditions. Images were displayed for 1 s at the beginning of each trial followed by a 500 ms fixation cross (see Figure 1A; gambling task, Simon and Go/No-go tasks) or balloon (BART). Images representing binge foods were designed to induce craving for binging (Sobik et al., 2005), relaxation (neutral stimuli) or stress (from the International Affective Picture System). We used 50% of neutral pictures to avoid a saturation of anxiety in patients (Lo Sauro et al., 2008), 25% of stress-inducing pictures and 25% of food pictures and presented them in a randomized order. Each picture was displayed only once across the experiment. Once all tasks have been completed, the anxiety produced by every image was assessed with on a continuous digital scale ranging from 0 to 100, 0 referred to an absence of anxiety; 100 to a life-threatening situation.

#### Data Collection

Weight was measured with a 0.1 kg precision and height with a 1 mm precision. Socio-demographic characteristics were collected with a self-administered questionnaire.

### Statistical Considerations

#### Decision Making Under Risk: The Gambling Task

First, we used the framework of the prospect theory to investigate participants’ loss aversion and their sensitivity to both gain and losses in the framework of prospect theory (Kahneman and Tversky, 1979; Lattimore et al., 1992; Tversky and Kahneman, 1992). According to this theory, individual choices are translated into model parameters that reflect several aspects of decision making under risk: loss aversion (parameter θ), sensitivity to the magnitude of the rewards (parameters β+ and β−, thus positive for gains and negative for losses), preference for risk (parameter δ) and a probability weighting function (parameter γ; see equation s1.1 in “Supplementary Material”). The corresponding model was fitted over each participant’s choice. Losses refer to the difference between a 0€ outcome to the gamble and the certain payoff.

#### Decision Making Under Uncertainty: BART

For each participant and each condition, we computed the rate of balloons saved, the average number of pumps per sequence and its standard deviation that captures intra-individual variability in each condition, and reaction time of each pump and each save for balloons saved. Likewise for the gambling task, we assessed loss aversion and sensitivity to gain and losses with an adapted model of the prospect theory to the repeated measures of the BART (Wallsten et al., 2005; see “Supplementary Material (Methods)”).

#### Simon and Go/No-Go Task

Average reaction time at correct incongruent trials (i.e., trials in which the side of the screen on which the arrow was displayed was opposite to the direction of the arrow) was computed in each condition. It measures the participant’s ability to process task irrelevant information. The rate of correct responses at go trials, error rate at no-go trials in the Go/No-go task and the interference effect in the Simon task (i.e., difference of average reaction time between correct incongruent and congruent trials) were computed in the neutral condition as control parameters of the decision process.

#### Statistical Analysis

Only participants who performed all tasks were included in the analyses (i.e., 16 ANR, 19 ANB, 18 BN and 18 controls).

The analyses were built in five main steps.

Step 1: we assessed participants’ performances in neutral condition for all tasks to make sure that the four groups had similar profiles. We performed ANOVAs when data were normally distributed and a Kruskal-Wallis test otherwise.

Step 2: we first compared the differences of behaviors between food and neutral conditions (reported as food-specific performances below) within each binging patients group. We then compared these differential behaviors between food and neutral conditions to those of each control group (ANR, healthy participants). These two later comparisons allowed identifying differences that would be specific to the binge and those that would be specific of binging and non-binging patients. We performed paired or two samples *t*-tests when data were normally distributed and Wilcoxon or Mann-Whitney tests otherwise. Reaction time at BART was analyzed with linear mixed models (see “Supplementary Material (Methods)”).

Step 3: we investigated whether the differential effects observed in step 2 were similar to the differential effects between stressful and neutral conditions (reported as stress-specific performances below) in patients. Such a comparison requires adjusting for the magnitude of the anxiety arousal to draw conclusions about the type of stimulus. In the BART task, we ran a linear mixed model in which the specific rates of balloons saved for food-specific and stress-specific conditions were modeled as a function of the following variables: (1) food-specific and stress-specific anxiety arousal; (2) condition (food/stress); (3) group (BN/ANR); (4) interaction between group and condition; (5) interaction between the group and the anxiety arousal; and (6) participants as random factor.

Step 4: this analysis complemented step 3. We investigated whether the differential effects observed in step 2 in patients were similar to those between stressful and neutral conditions in healthy participants. At the BART task, we tested whether BN and ANR patients primed with food cues and healthy participants primes with stressful cues behaved similarly independently of the magnitude of the anxiety aroused by food and stressful cues. The specific rates of balloons saved in the food condition for BN and ANR patients and in the stress condition for healthy participants were modeled as a function of: (1) anxiety arousal; and (2) group (BN/ANR/healthy controls).

Note: we ran the same models of steps 3 and 4 for the average number of pumps and average reaction time when inflating a balloon at the BART task. For the standard deviation of the number of pumps per balloon, BN and ANR were replaced by ANB patients only. We also conducted steps 3 and 4 for the gambling task. The dependent variable was then loss aversion, relative bias toward the sure option and the rate of choices of the certain payoff when the probability to win the gamble was equal to 50%. For the gambling task, ANB patients were added to BN and ANR for loss aversion and the rate of choices of the certain payoff when probability to win the gamble as equal to 50%. For the relative bias toward the sure option, only ANB patients were included.

Step 5: we investigated whether the differences observed in step 2 between ANB patients on the one side and ANR and BN on the other side were due to an interfering cognitive conflict that would recruit some cognitive resources and hold back patient from performing optimally at the BART. The rationale of this step relies on previous results that showed that ANB patients actively avoid binging while binge food cues are priming them for the binge, in contrast with BN and ANR patients who do not experience such a conflict (Neveu et al., 2014).

We modeled the standard deviation of number of inflates for balloons won specific to the food condition (i.e., differences between food and neutral conditions) as a function of: (1) the average reaction time at correct incongruent trials at the Simon task; (2) group (ANB/BN+ANR); and (3) their interaction (model 1).

All tests were two tailed. *P*-values were corrected for multiple testing using the Benjamini-Hochberg correction (Benjamini et al., 2006). Analyses were carried out with R software (release 3.0.1) and Matlab (release 2011a, Mathworks Inc) using *lme4* R package for model estimates.

## Results

### General Group Characteristics

The four groups of participants had similar profiles with respect to their educational level, source of financial income, father’s socio-professional status, inhibitory control, and capacity to ignore goal irrelevant information (Table 1 and “Supplementary Table 1”). ANR patients were younger than the others (*p* = 0.05, Table 1). However, age did not correlate with any of the food specific parameters extracted of the BART and the gambling task (see “Supplementary Material (Results)”).

**Table 1:** **Socio demographic, behavioral (neutral condition) and physiological characteristics for the four groups**.

Socio-demographic characteristics	*n*	Bulimia nervosa	*n*	Controls	*n*	Anorexia nervosa binging subtype	*n*	Anorexia nervosa restrictive subtype	*p*-value
Age, year	18	24.2 (5.78)	18	24.3 (3.21)	19	25.6 (4.92)	16	21.7 (5.04)	0.046
Educational level, years	14	12.8 (2.23)	18	14.2 (2.43)	14	14.1 (2.96)	16	13.3 (1.59)	0.2
Source of financial income	16		17		14		15		0.33
With own income, *n*(%)		9 (56.3)		8 (47.1)		7 (50)		3 (20)	
With parental financial support, *n*(%)		6 (37.5)		7 (41.2)		6 (42.9)		11 (73.3)	
With fellowship, *n*(%)		0 (0)		2 (11.8)		0 (0)		1 (6.67)	
Other, *n*(%)		1 (6.25)		0 (0)		1 (7.14)		0 (0)	
Paternal socio-professional status, *n*(%)	15		18		15		13		0.5
INSEE 1 or 2		1 (6.67)		2 (11.1)		2 (13.3)		2 (15.4)	
INSEE 3		5 (33.3)		6 (33.3)		6 (40)		7 (53.8)	
INSEE 4, 5 or 6		6 (40)		8 (44.4)		3 (20)		4 (30.8)	
INSEE 7 or 8		3 (20)		2 (11.1)		4 (26.7)		0 (0)	

**Behavioral and physiological characteristics**		**Bulimia nervosa (*n* = 18)**		**Controls (*n* = 18)**	***p*-value**	**Anorexia nervosa binging subtype (*n* = 19)**		**Anorexia nervosa restrictive subtype (*n* = 16)**	***p*-value**

Error rate at no-go trials, %		2.2 (4.6)		4.8 (10.2)	0.48	2.8 (5.6)		2.1 (4.0)	0.88
Rate of good responses at go trials, %		100 (0)		99.8 (0.5)	0.34	100 (0)		99.7 (0.8)	0.13
Error effect* at Simon task, %		−2.4 (4.4)		−4.0 (5.5)	0.42	−5.9 (9.2)		−9.1 (21)	0.57
Interference effect** at Simon task, ms		63 (77)		60 (60)	0.44	69 (73)		44 (46)	0.44
Body Mass Index (kg/m^2^)		20.6 (3.19)		21.5 (3)	0.19	17.6 (1.84)		15.3 (1.19)	0.0003
Duration between last meal
and BART assessment, mn		73.4 (137)		154 (43.5)	0.33	105 (140)		96.8 (141)	0.82

Mean (standard deviation) are reported for quantitative parameters. INSEE ^#^socio-professional category according to the “Institut National de la Statistique et des Etudes Economiques”: 1,2: Farmer, craftsman, shopkeeper and large retailer, chairman and managing director; 3: senior executive, manager; 4,5,6: intermediate jobs, employees and workers; 7,8: retired, without any job. *Error effect, rate of error for incongruent trials−rate of error for congruent trials. **Interference effect, reaction time for incongruent trials−reaction time for congruent trials.

Stressful images elicited higher anxiety than neutral images in all four groups (“Supplementary Figure 1”). On the other hand, food images aroused higher anxiety compared to neutral images in ANR, ANB and BN patients only (“Supplementary Figure 1”).

In the neutral condition, the four groups showed similar decision profiles under risk and under uncertainty (see “Supplementary Material (Results)”).

### Comparison between Food and Neutral Conditions

#### Decision Making Under Uncertainty: The BART

**Figure 2:**
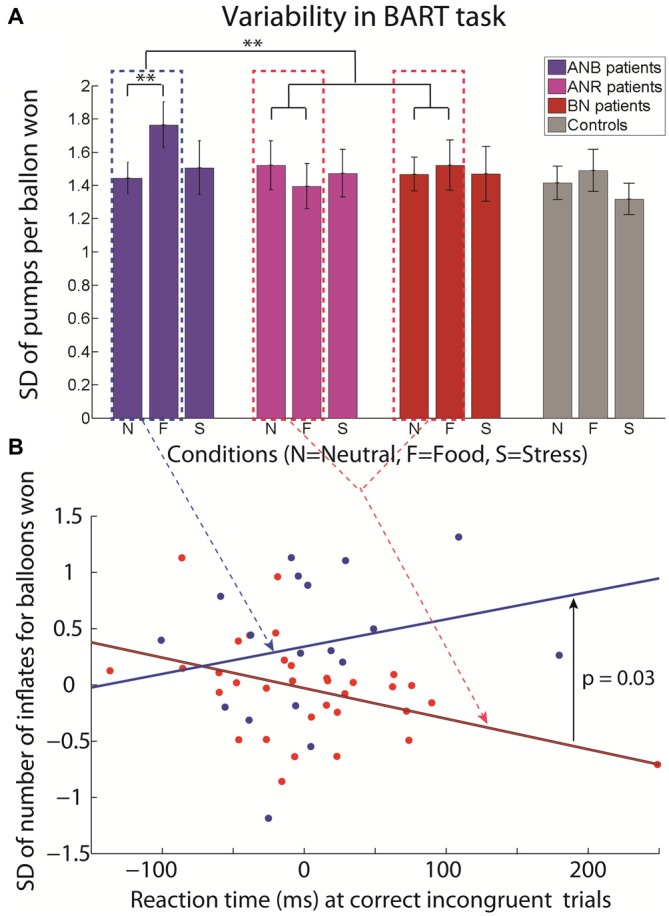
**Standard deviation (SD) of the number of inflates per balloons won in BART in ANR subtype, ANB subtype, BN and Healthy controls in neutral, food and stressful conditions (A); and association between the difference in reaction time at correct incongruent trials in Simon task for the binge food and neutral conditions and the difference in the standard deviation of number of inflates per balloon won in the BART in binge food and neutral conditions for BN+ANR patients (red) and ANB patients (blue; B).** Mean and standard errors of the mean are reported for **(A)**. Note: **p < 0.05.

BN patients exhibited more conservative behaviors in food compared to neutral conditions: they saved more balloons (*p* = 0.03, Figure 1B) and pumped less balloons in a balloon sequence (*p* = 0.001, Figure 1B). In contrast, ANB patients did not (*p* > 0.1, Figure 1B). The difference in rate of balloons saved between food and neutral conditions was similar in BN and ANR patients (*p* = 0.51, Figure 1B) as well as between ANB and healthy participants (*p* = 0.88, Figure 1B). Similar results were obtained with the average number of pumps (*p* = 0.63 and *p* = 0.46 respectively, Figure 1B). However, only ANB patients exhibited a higher variability in the number of pumps for balloons saved across sequences when they were primed by food image as compared with neutral images (mean standard deviation of number of inflates for balloons saved (SD): 1.76 (0.60) in the food vs. 1.44 (0.41) in neutral condition, *p* = 0.04, Figure 2A). The difference between food and neutral conditions in this latter parameter was significantly more associated with the difference between food and neutral conditions in the reaction time at correct incongruent trials in ANB patients than in BN and ANR patients (β = 0.0052, *p* = 0.03, model 6, Figure 2B, model 1).

The pattern of differences in reaction time when inflating a balloon between food and neutral conditions in the four groups was consistent with the results obtained with the rate of balloons saved and average number of inflates (“Supplementary Material (Results)”).

Additionally, the parameters of the decision process investigated in the framework of Prospect Theory were similar in food and neutral conditions in binging patients (“Supplementary Material (Results)”).

#### Decision Making Under Risk: the Gambling Task

When participants were primed with food as compared with neutral cues, binging patients selected more often the sure payoff option than the gamble when the probability to win was set at 50% (mean difference between food and neutral conditions (SD): 13.9% (34.2%), *p* = 0.02, Figure 1C). This proportion was higher than in healthy participants (mean (SD): 13.9% (34.2%) vs. 0.6% (32.1%), *p* = 0.04) but similar to the one of ANR patients (mean (SD): 13.9% (34.2%) vs. 9.2% (25.3%), *p* = 0.63). Coherently, binging and ANR patients expressed a similar difference in aversion to losses between food and neutral conditions (*p* = 0.97) and aversion to losses was higher in food than in neutral conditions (median (interquartile): 1.06 [0–7.9] in food condition vs. 0.46 [0–0.74] in neutral condition, *p* = 0.009). Preference for risk and the sensitivity to gains and losses were similar between the two conditions in binging patients (“Supplementary Material (Results)”).

### Comparison between Food-Specific Behavior and Stress-Specific Behavior in Patients

After adjusting for the magnitude of anxiety, we found that BN and ANR patients inflated less balloons in the BART task when exposed to food cues as compared to stressful cues (“Supplementary Table 1”). Consistently, all patients chose more often the safe option in the gambling task when exposed to food cues as compared to stressful cues (“Supplementary Table 1”).

### Comparison between Patients’ Food-Specific Behavior and Healthy Participants’ Stress-Specific Behavior

After adjusting for the magnitude of anxiety, we found that the difference between food and neutral conditions in the two tasks in patients was similar to the difference between stressful and neutral conditions in healthy participants (“Supplementary Table 2”).

## Discussion

In this study, BN patients exhibited more conservative behaviors under risk and under uncertainty and consistently had a higher loss aversion under risk and a lower sensitivity to gains under uncertainty after being primed by binge food cues compared to neutral cues. ANB patients also exhibited more conservative behaviors under risk but did not under uncertainty. Interestingly, at the BART, they showed a higher variation in the number of pumps after being primed by food cues compared to neutral cues. This difference in variation of pumps between food and neutral conditions was associated with a difficulty to process information irrelevant to the task. We also showed that BN patients exhibited similar conservative behaviors under risk and under uncertainty in food and stressful conditions. Additionally, these more conservative behaviors in food condition in BN patients were similar to those of non-binging patients (ANR) after being primed by food cues and of healthy participants after being primed by stressful cues.

These results suggest that situations engaging food that is perceived as threatening by patients with eating disorders (Fairburn and Harrison, 2003) caused patients to adopt more conservative behaviors. Indeed, compared to neutral cues, food stimuli triggered more conservative choices in the BART and the gambling task, consistently with a higher aversion to losses, in binging and non-binging patients. Such a behavior might suggest that the decision process might be modulated towards safe options to prevent losing the cumulated efforts performed to restrict food intake since the last binge. Indeed, exposure to foods and especially binge foods, that are usually highly palatable (Walsh et al., 1989), increases binge craving in binging patients (Sobik et al., 2005), and thus threatens all the effort it took in the strict control of food intake. This mechanism is supported by the higher anxiety elicited by food cues as compared to neutral cues observed in our patients. This prevention of a loss of the effects of the effort to restrict food intake would be implemented through an active avoidance of the binge or through an over-intake of food during the binge to reduce food attractiveness and facilitate strict dieting thereafter as previously shown with the same patients (Neveu et al., 2014).

The fact that ANB patients did not exhibit more conservative behaviors in food compared to neutral conditions at the BART does not invalidate this view. Contrary to BN patients, they might have been engaged in an active avoidance of the binge while food cues were priming them for the binge (Neveu et al., 2014). This cognitive conflict would have interfered with the task. This assumption is supported by the ANB’ higher variability in the number of pumps in the BART in food than in neutral conditions, and the positive association between the variability of the number of pumps in the BART and the difficulty to process task-irrelevant information.

Aligned with previous work, our results support the view that the decision process in patients with eating disorders is not fundamentally altered (Cavedini et al., 2004; Van den Eynde et al., 2012; Wu et al., 2013; Neveu et al., 2014). First, compared to a neutral setting, binge food exposure was associated with safer and more conservative behaviors in patients. When this behavior was not exhibited, patients were facing a cognitive conflict irrelevant to the decision, biasing their decision process. This finding suggests that patients adapt their decision to the context that they encounter. Second, the evidence of more conservative behavior in food and stressful conditions in patients suggests that this particular behavior is mainly due to stress level and not to a general impairment. Third, the patients’ propensity to exhibit more conservative behaviors when primed by food cues (i.e., a stressful situation for them) was similar to the one of healthy participants in a stressful environment (when primed by stressful pictures) and to previously reported results in healthy women (Lighthall et al., 2009; Porcelli and Delgado, 2009; van den Bos et al., 2009; Starcke et al., 2011).

To sum up, binging patients’ decision process under risk and uncertainty is modulated toward safe options when patients are primed by binge foods cues as it is under uncertainty in healthy participants when primed by stressful cues. However, an active avoidance of the binge would interfere with the decision process and would reduce the promotion of safe choices in a binge situation. This interference would likely favor binge occurrence by narrowing the patient’s view of the situation (Harrison et al., 2011) through a limited availability of cognitive resources at the time of decision.

These results have several implications. First they might explain the discrepancies observed in previous studies. Indeed, the previously reported differences between binging and healthy participants in a neutral setting (Brand et al., 2007; Tchanturia et al., 2007; Brogan et al., 2010) might be the result of a different goal at the time of assessment, such as an active avoidance of the binge that would interfere with the task. Thus, claims about decision-making impairments in binging patients should be tuned down. Second, the difference in conservative behavior between conditions suggests that therapeutic interventions targeting the decision process to improve binge eating might be poorly efficient. Indeed, patients might switch goal at any time: from setting the binge to an active avoidance of it (Fairburn and Harrison, 2003; Neveu et al., 2014). Moreover, adopting safer behaviors is not specific to the binge, as previously reported (Guillaume et al., 2015), nor of the food condition. Third, our results highlight the limit of relying only on direct comparisons to make assumptions about symptoms and rather show the importance of using differential measures when investigating differences between healthy participants and patients groups. An experimental condition using ecological cues such as food cues for binging patients should always be compared to a neutral one to make assumptions about symptoms, contrary to what is usually done (Cavedini et al., 2004; Brand et al., 2007; Tchanturia et al., 2007; Brogan et al., 2010; Van den Eynde et al., 2012; Wu et al., 2013). The interference with other tasks at the time of assessment, like the active avoidance of the binge, might be a major confound.

Our results should however be interpreted carefully. Patients included in the study were hospitalized and under medication. However, we controlled for the effect of medication by including in the study only those patients for whom medication had been stabilized for more than 1 week before assessment (van Laar et al., 2002; Drueke et al., 2009). Second, the selection bias related to the recruitment of inpatients is limited. Hospitalization is a recommended setting for cares in AN and over 50% of BN patients experienced at least one hospitalization (Spindler and Milos, 2004; American Psychiatric Association, 2006). We also reproduced several previously published results in these populations (Cavedini et al., 2004; Van den Eynde et al., 2012; Chan et al., 2013; Wu et al., 2013) and in healthy controls (Lighthall et al., 2009; Porcelli and Delgado, 2009; van den Bos et al., 2009; Starcke et al., 2011). Finally, results cannot be generalized to male eating disorder patients because of unpredictable differences between men and women (Lighthall et al., 2009; van den Bos et al., 2009).

## Conclusion

The adjustment of eating disorder patients’ decision process to the current context, and the interference caused by the active avoidance of the binge previously shown in the same participants (Neveu et al., 2014), suggest that studies investigating cognitive processes in these patients should be conducted according to a new framework. These studies should systematically use food cues and assess patient’s current disposition toward foods. This will avoid serious confounding factors and provide a better understanding of how patients dynamically recruit cognitive skills according to the current context.

## Author Contributions

RN, DN, GC and AN designed the experiment. RN, FB, EF, EC and ML collected data. RN, DN, EF and GC analyzed the data. RN, EF, GC and DN drafted the manuscript. FB, EC, ML and AN critically reviewed the manuscript.

## Funding

This work was financially supported by the European Research Council (ERC Consolidator Grant 617629) grant, Praxis and CNRS.

## Conflict of Interest Statement

The authors declare that the research was conducted in the absence of any commercial or financial relationships that could be construed as a potential conflict of interest. The reviewer FB and handling Editor declared their shared affiliation, and the handling Editor states that the process nevertheless met the standards of a fair and objective review.
