# Supplementary material for: Preference for Safe Over Risky Options in Binge Eating
Source: Front Behav Neurosci. 2016 Mar 31;10:65. doi: 10.3389/fnbeh.2016.00065 (PMC4815053; doi:10.3389/fnbeh.2016.00065)
Supplement: Supplementary file 1 [file Presentation_1.pdf]

Supplementary methods

Selection of images of binge foods

Binge foods images were selected from the lists of foods ingested during binges established by two BN patients who did not perform the tasks. These lists were sorted by increasing order of craving induction for the binge. One patient was more attracted by salty foods and the other one by sweeter foods.

Fitting equation s1.1 over choices of the gambling task

$$Percieved\ outcome = \frac{\delta * p^{\gamma}}{\delta * p^{\gamma} + (1-p)^{\gamma}} * gain^{\beta+} - \theta * \left(1 - \frac{\delta * p^{\gamma}}{\delta * p^{\gamma} + (1-p)^{\gamma}}\right) * loss^{\beta-}$$ (Equation s1.1)

Gains and losses of gambles are computed according to the sure payoff. Indeed, if a participant has to choose between 1€ for sure and 4€ with 25% of chances to get it, choosing the gamble will lead to 0€ in 75% of cases and thus to a relative loss of 1€ compared to the sure payoff. Similarly, in 25% of cases, it will lead to a 3€ gain. We therefore computed gains as the difference between the gamble payoff in case of winning and the sure payoff. Losses were set to the amount of the certain payoff.

Participants would therefore choose the gamble if the perceived payoff computed by equation 1 is higher than the certain payoff proposed. We therefore fitted a logistic model over participants' choices following the same reasoning as for the hyperbolic discounting function:

Proba (choice = gamble option) = e^a/ (1 + e^a) (Equation s1.2)

Proba (choice = sure option) = 1 / (1 + e^a) (Equation s1.3)

With a = perceived payoff - certain payoff and perceived payoff defined by equation s1.1.

For each participant, parameters were estimated by maximizing the log likelihood (i.e. minimizing – log(likelihood)) with matlab fminsearch function the error between fit and participant's choice. In order to avoid to fall into a local minimum when running the fminsearch function, we run it for all combinations of initializations among a set of 3 different initializations for each parameter of equation 1: 0.1, 0.5 and 1. The fit with the highest log likelihood was selected.

Fitting Wallsten model over participants' choices at BART

Wallsten & al. proposed a derivative model of equation s1.1. This model includes the same parameters of aversion to losses, sensitivity to gains and losses as in equation s1.1 but also adds a learning parameter that take into account the learning of probabilities for each balloon to explode across balloons. However, compared to equation s1.1, perception of probabilities are replaced by a model of the estimation process made by participants of the probability of balloons to explode based on the number of inflates done in the previous balloons. This leads to equation s1.4:

$$Percieved\ outcome\ at\ pump\ i = \rho * (p_i * x^{\beta+} - \theta * (1-p_i) * ((i-1)*x)^{\beta-})$$ (equation s1.4)

Where β+ and β- measures the sensitivity to the magnitude of the reward (either positive for gains or negative for losses), θ, aversion to losses, ρ, learning rate, i the number of pumps, x, the quantum of money won at each inflate, and pi represents the probability for the balloon to explode at pump i. As the learning process occurs across all balloons and we compared conditions between them, the estimate of pi by each participant based on the previous number of inflates done for the previous balloons and on participants a priori before the first balloon would be the same for each condition. Therefore, we replaced this model of probabilities estimate by the true probability for the balloon to explode at pump i. This limits non linearities in equation s1.4 and therefore leads to a more reliable estimation of parameters of equation s1.4.

Parameters of equation s1.2 were estimated by maximizing the likelihood of a logistic model using matlab fminsearch function as for the gambling task:

Proba (choice = pumping at inflate i) = $e^{\text{percieved outcome at pump i}}$/ (1 + $e^{\text{percieved outcome at pump i}}$) (Equation s1.5)

Proba (choice =save balloon at pump i) = 1 / (1 + $e^{\text{percieved outcome at pump i}}$) (Equation s1.6)

In order to avoid to fall into a local minimum when running the fminsearch function, we run it for all combinations of initializations among a set of 4 different initializations for each parameter of equation 1: 0.1, 1 and 2 for β+, β- and θ, and 0.1, 1 and 1.5 for ρ. The fit with the highest log likelihood was selected.

Reaction time at BART

A linear mixed model was carried out first within each group of binging participant. Reaction time was modeled as a function of the condition (Food/Neutral/Stress), type of choice (pump/save balloon), their interaction and participants as a random factor over the intercept. The stressful condition was added to improve the estimate of the variance of each condition. Second, a linear mixed model was carried out between binging participants and ANR.

Supplementary results:

Neutral condition

At the BART task, the rate of balloons saved (p=0.87, figure 1B), average number of pumps (p=0.55, supplementary figure 3) and standard deviation of the number of pumps per balloon (p=0.94, figure 1B) were similar. The rate of certain payoff when the gamble probability is at 50% at the gambling task (p=0.73, figure 1C), loss aversion in the gambling task (p=0.46, figure 3) and in the BART (p=0.46), perception of gains and losses in gambling task (p=0.63 and p=0.48 respectively, figure 3) and in the BART (p=0.33 and p=0.35 respectively), decision making duration computed by the DDM (p=0.87) and relative bias toward the sure option at gambling task (p=0.64) were also similar between the four groups.

Association between age and food specific parameters of the two tasks

Age did not correlate with the food specific rate of balloons saved, average number of pumps per balloon won, standard deviation of number of pumps per balloon won at the BART and rate of choices of the safe option at the gambling task in ANR and ANB patients ($r_{ANR+ANB}$=0.1, p=0.58; $r_{ANR+ANB}$=-0.13, p=0.47; $r_{ANR+ANB}$=-0.08, p=0.64; $r_{ANR+ANB}$=0.03, p=0.86; respectively).

BART task

BN and ANR patients were slower to inflate a balloon in food compared to neutral condition (mean difference (standard error of the mean (SEM)): 41ms (22.8ms), p=0.07 and 75.6ms (20.7ms), p=0.0003 respectively) while ANB patients had a similar reaction time (mean difference (SEM): -12.2ms (20ms), p=0.54). Reaction time to save a balloon was higher than to inflate it in all patients in neutral condition (mean difference (SEM): 165.7ms (25.7ms), p<0.0001 in BN; 276.3ms (26.3ms), p<0.0001 in ANR; 287.8ms (25.3ms), p<0.0001 in ANB). Reaction time to save a balloon was higher in food than in neutral condition only in ANR patients (mean difference (SEM): 66.2ms (38.1ms), p=0.08).

The aversion to losses was similar in food and neutral conditions in BN (median difference between food and neutral conditions (interquartile): -0.11 [-1.64 – 0.97], p=0.78) and ANB patients (median difference between food and neutral conditions (interquartile): 0.02 [-0.42 – 0.88], p=0.28). Similar results were obtained for the weighting of gains and losses (median difference between food and neutral conditions (interquartile): 0.07 [-0.36 – 0.6], p=0.81 for β+ in BN patients and 0.17 [-0.3 – 0.83], p=0.12 in ANB patients; -0.0003 [-0.47 – 0.39], p=0.91 for β- in BN patients and 0.003 [-0.12 – 0.73], p=0.24 in ANB patients).

Gambling task

BN and ANB patients had a similar sensitivity to gain and losses in food and neutral conditions (median difference between food and neutral conditions (interquartile): -0.27 [-1.2 – 1.7], p=0.99 for β+ and 0.37 [-2 – 1.3], p=0.6 for β-) as well as a similar sensitivity to risk (median difference between food and neutral conditions (interquartile): -0.04 [-0.49 – 0.03], p=0.24).

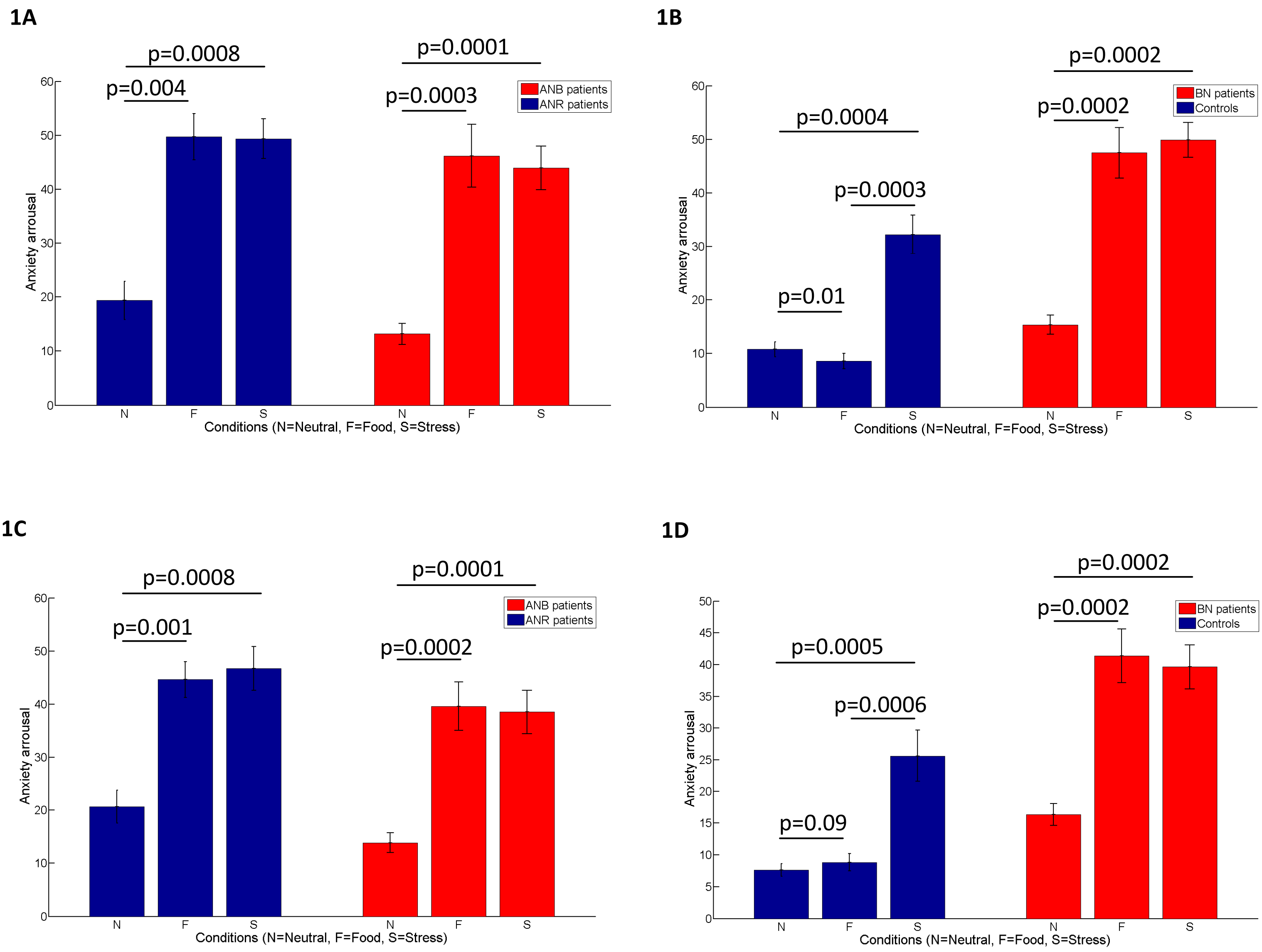

Supplementary figure 1: emotional arousal rated from 0 (no emotion) to 100 (highest emotion ever felt) generated by images displayed during the BART task (figures 1a and 1b) and gambling task (figures 1c and 1d) in bulimia nervosa (BN), anorexia nervosa binging subtype (ANB) and anorexia nervosa restrictive subtype (ANR) patients and healthy controls. Mean and standard errors of the mean are reported. Only p-values < 0.1 are reported.

Task	Dependant variable	Group	Beta estimate (SEM)	F	p-value
BART	Rate of balloons saved, %			0.15	0.7
		ANR	-2.55 (2.51)		0.32
		BN	1.2 (2.38)		0.62
BART	Mean number of inflates			6.26	0.02
		ANR	0.45 (0.26)		0.09
		BN	0.45 (0.25)		0.08
BART	Reaction time to inflate a balloon, ms			1.6	0.22
		ANR	26.5 (59)		0.66
		BN	76.5 (55.9)		0.18
BART	SD of number of inflates	ANB	-0.24 (0.18)	NA	0.19
Gambling task	Rate of choices of the safe option when p=50% to win, %			8.31	0.006
		ANR	-13.3 (8.99)		0.15
		ANB	-11 (8.24)		0.19
		BN	-18.5 (8.47)		0.04
Gambling task	Aversion to loss			0.002	0.97
		ANR	-11 (116)		0.93
		ANB	158 (107)		0.15
		BN	-138 (110)		0.21

Supplementary table 1: Parameter estimates of the differences between food specific and stress specific differences within patients for the parameters of the BART and the gambling tasks that exhibited differences between neutral and food conditions. Mean (Standard Error of the Mean, (SEM)) are reported for the estimates of the difference between stress specific (difference between stressful and neutral conditions) and food specific (difference between food and neutral conditions) differences for the dependant variable after regressing out the magnitude of the anxiety. A positive value means that the difference is stronger for the stress specific difference.

Note: *NA=Not Applicable; ANR=Anorexia Nervosa Restrictive subtype; ANB=Anorexia Nervosa Binging subtype; BN=Bulimia Nervosa*

Task	Dependant variable	Group	Beta estimate (SEM)	F	p-value
BART	Rate of balloons saved, %			0.62	0.54
		ANR	2.97 (3.63)		0.42
		BN	0.36 (0.92)		0.92
BART	Mean number of inflates			2.16	0.13
		ANR	-0.47 (0.38)		0.22
		BN	-0.63 (0.37)		0.1
BART	Reaction time to inflate a balloon, ms			1.71	0.19
		ANR	63.7 (40.3)		0.12
		BN	28.6 (39.5)		0.47
BART	SD of number of inflates	ANB	-0.37 (0.2)	NA	0.07
Gambling task	Rate of choices of the safe option when p=50% to win, %			1.38	0.26
		ANR	11 (9.5)		0.25
		ANB	14.6 (9.2)		0.12
		BN	17.7 (9.3)		0.06
Gambling task	Aversion to loss			0.35	0.79
		ANR	-15 (137)		0.91
		ANB	6.8 (132)		0.96
		BN	108 (134)		0.42

Supplementary table 2: Parameter estimates of the differences between food specific food difference in patients and stress specific difference in healthy participants for the parameters of the BART and the gambling tasks that exhibited differences between neutral and food conditions. Mean (Standard Error of the Mean, (SEM)) are reported for the estimates of the difference between patients and healthy participants after regressing out the magnitude of the anxiety. A positive value means that the difference is stronger in patients than in healthy participants.

<u>Note:</u> NA=Not Applicable; ANR=Anorexia Nervosa Restrictive subtype; ANB=Anorexia Nervosa Binging subtype; BN=Bulimia Nervosa
